# Mirizzi Syndrome

**DOI:** 10.1155/1988/54294

**Published:** 1988

**Authors:** Thomas C. Bower, David M. Nagorney

**Affiliations:** Department of Surgery Mayo Clinic 200 First Street SW Rochester Minnesota 55905 USA

## Abstract

The Mirizzi syndrome refers to benign obstruction of the common hepatic duct by a stone impacted within the neck or cystic duct of the gallbladder, which causes extrinsic compression of the common hepatic duct and obstructive jaundice. Although a rare cause of obstructive jaundice, it remains a clinically and surgically challenging problem. Five patients with the Mirizzi syndrome were culled from over 9000 patients undergoing operation for gallstone disease. The management of these patients was detailed. Diagnosis requires a high index of clinical suspicion but can be confirmed with the use of ultrasonography and percutaneous transhepatic cholangiography. Cholecystectomy and common duct exploration are essential components of operative therapy, but additional procedures to repair non-circumferential bile duct defects or strictures must be anticipated.

## INTRODUCTION

Benign mechanical obstruction of the common hepatic duct by a stone impacted in the cystic duct or neck of the gallbladder or by the concomitant inflammation is a relatively uncommon cause of obstructuve jaundice. This type of partial mechanical obstruction of the common hepatic duct by a cystic duct stone was initially described by Kehr in 1905[1] and Ruge in 1908[2]. In 1948, Mirizzi[3] subsequently described a "functional hepatic syndrome" in patients with jaundice caused by an inflammatory reaction from a stone impacted in the cystic duct or neck of the gallbladder. Although he erroneously postulated that the inflammation resulted in spasm of a physiologic and anatomic sphincter comprised of circular muscle fibers within the common hepatic duct, this clinical entity has been currently coined as the "Mirizzi syndrome".

Our purpose is to review our recent experience with this uncommon clinical syndrome and further define the management of these patients.

## PATIENTS AND METHODS

Five patients with Mirizzi syndrome have been encountered at the Mayo Clinic between 1976 and 1986. We included only those patients with mechanical compression of the common hepatic duct by gallstones with associated acute or chronic inflammation of the gallbladder and a history of jaundice or cholangitis that was specifically recognized as benign, extrinsic bile duct obstruction by the surgeon intra-operatively. During the study period, 8220 cholecystectomies and 1228 common bile duct explorations were performed. Patient demographics, clinical features, laboratory data, results of diagnostic studies, and operative findings were obtained from patient records. Follow-up was complete in four of five patients and ranged from 12 to 130 months. One patient was lost to follow-up.

## FINDINGS

The clinical characteristics and laboratory data of our patients are outlined in Table 1. Laboratory findings were compatible with obstructive cholestasis without acute inflammation in four of five patients.

**Table 1**

| *Age* | *Sex* | *Symptoms* | *Laboratory data* | | | |
|---|---|---|---|---|---|---|
| | | | *WBC* *(× $10^3$)* | *Alk Phos* *(units/l)* | *SGOT* *(units/l)* | *T. bili* *(mg/dl)* |
| 66 | F | History jaundice, pruritus, anorexia | 5.1 | 1148 | 60 | 2.0 |
| 77 | F | Jaundice, RUQ pain, fatigue, weight loss | 5.4 | 1284 | 39 | 9.0 |
| 46 | F | Jaundice, fever, chills, pruritus | 7.7 | 887 | 68 | 17.8 |
| 71 | F | RUQ pain | 8.6 | 252 | 18 | 0.6 |
| 56 | M | Jaundice, pruritus, anorexia | 6.2 | 659 | 125 | 11.0 |

### *Radiologic Studies*

Preoperative diagnostic studies included intravenous cholangiography (IVC), abdominal ultrasonography and percutaneous transhepatic cholangiography. IVC was performed in only one patient and showed a gallstone without visualization of the biliary tree. Since 1979, ultrasonography and percutaneous transhepatic cholangiography have been employed for diagnosis. Ultrasonography showed a thick-walled gallbladder containing one or more large gallstones and dilated intrahepatic and extrahepatic ducts (Figures 1a and 1b). The Mirizzi syndrome was diagnosed pre-operatively in only one patient by ultrasonography. Percutaneous transhepatic cholangiography showed dilatation of the intrahepatic and extrahepatic bile ducts proximal to the obstruction. The site of obstruction of the common hepatic duct was marked by a smooth eccentric curvilinear narrowing with a normal distal bile duct (Figure 2).

### *Operative Findings*

Intra-operatively, each patient had marked thickening and induration of the gallbladder with a dense ligenous reaction between the gallbladder and bile duct.

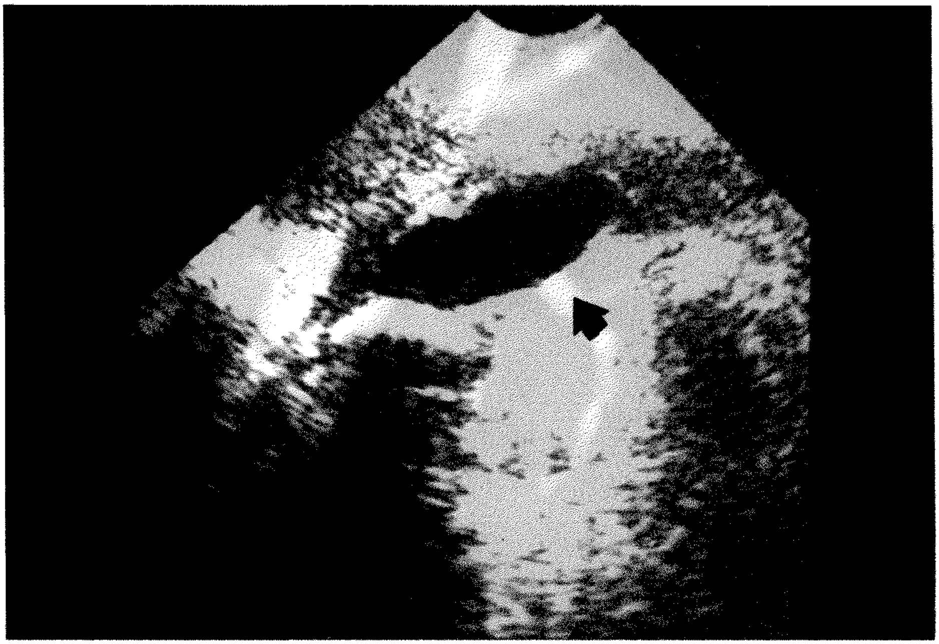

**Figure 1a** Abdominal sonogram showing thick-walled gallbladder (arrow) and large gallstone (arrowhead) within the neck and cystic duct of the gallbladder.

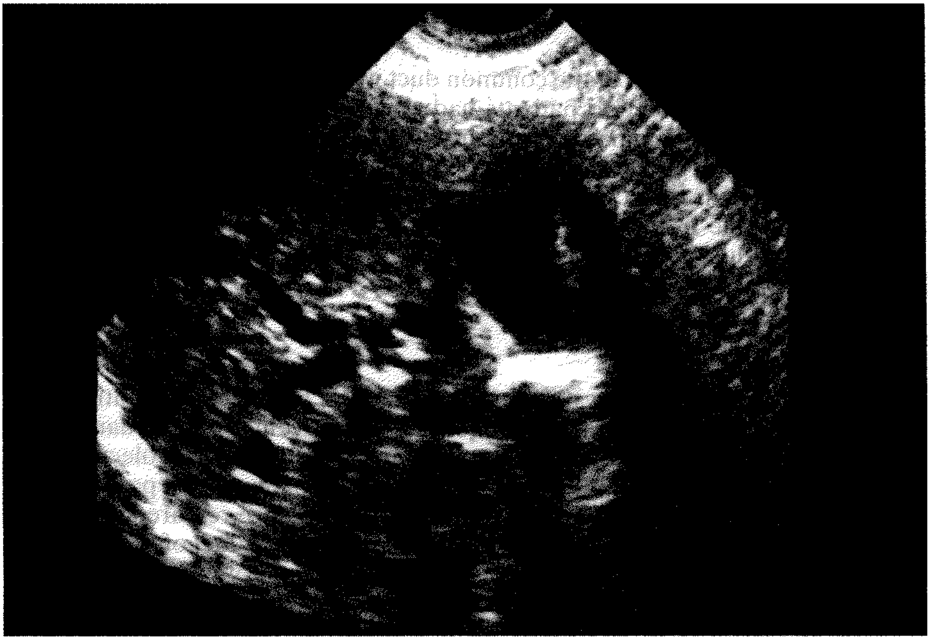

**Figure 1b** Abdominal sonogram showing dilated intrahepatic ducts (small arrow) and common hepatic duct to the level of the gallstone (arrowhead) within the neck of the gallbladder (arrow).

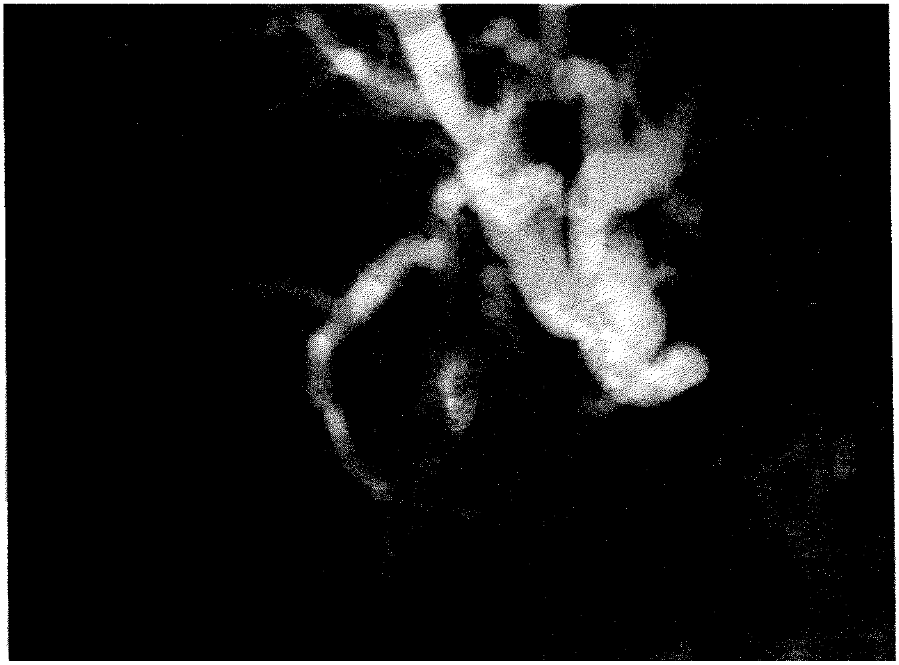

**Figure 2** Percutaneous transhepatic cholangiogram demonstrating the smooth medial bowing of the common hepatic duct (small arrow) with dilated intrahepatic ducts above (large arrow) and partial filling of common bile duct below (white arrow).

Obstruction or compression of the common duct was caused by stones in Hartman's pouch of the gallbladder. Two patients had cholecystohepatic duct fistulas, and a third patient had an inflammatory stricture of the common hepatic duct caused by inflammation by a large gallstone. Two patients had choledocholithiasis ranging from 0.5–1.7 cm. The exact relationship of the cystic duct to the common hepatic duct was discernible in two patients; one had a parallel (>3.0 cm) course to the common hepatic duct and one had a short wide (2.5 cm) cystic duct.

## TREATMENT

All patients underwent cholecystectomy and common bile duct exploration (Table 2). To better define biliary tract anatomy and prevent ductual damage, the gallbladder was often opened and the stones extracted prior to cholecystectomy. Concurrent common duct stones were extracted in two patients. All patients had T-tube choledochostomy. Cholecysto-hepatic duct fistulas were repaired with a patch technique utilizing a contiguous portion of the neck or cystic duct of the gallbladder in two patients. The patient with the inflammatory stricture of the common hepatic duct was bypassed with a Roux-en-Y choledochojejunostomy.

There was no operative mortality or morbidity. Complete resolution of the obstructive cholestasis, based on liver function tests, occurred in four patients. One patient had no follow-up liver function tests. Postoperative T-tube cholangiograms

**Table 2**

| *Patient* | *Radiologic studies* | *Operative findings (all with marked thickening of gallbladder)* | *Treatment* |
|---|---|---|---|
| 1 | Intravenous cholangiography | Acute/chronic cholecystitis (ligneous reaction), cholecysto-right hepatic duct fistula (3 stones in gallbladder <2.5 cm) | Cholecystectomy, common bile duct exploration, repair R. hepatic duct over T-tube with remnant of gallbladder tissue |
| 2 | Ultrasonography PTHC | Chronic cholecystitis (ligneous reaction), erosion of gallstone into hepatic duct bifurcation (1 stone 3.5 cm) | Cholecystectomy, extraction of stone, common bile duct exploration, repair of defect in hepatic ducts with remnant of gallbladder |
| 3 | Ultrasonography PTHC | Chronic cholecystitis, choledocholithiasis (ligneous reaction), erosion of stones into lateral wall of common bile duct with inflammatory stricture of common bile duct (2 gallstones 1.7 cm each, 1 common bile duct stone 1.7 cm) | Cholecystectomy, biopsy of common bile duct, common bile duct exploration, Roux-Y choledochojejunostomy |
| 4 | Oral cholecystogram with tomograms | Subacute cholecystitis (ligneous reaction), choledocholithiasis (multiple stones 0.5–2.5 cm, cystic duct and common bile duct 2.5 cm), common bile duct inflamed | Cholecystectomy, common bile duct exploration |
| 5 | Ultrasonography PTHC | Subacute and chronic cholecystitis, compression by stone of common hepatic duct, parallel cystic duct (3 cm) to common hepatic duct, (1 stone 4.5 cm) | Cholecystectomy, common bile duct exploration, T-tube choledochostomy |

were normal in four patients, but the patient with the cholecysto-right hepatic duct fistula had slight residual narrowing of the right hepatic duct at the ductal confluence, which was asymptomatic.

## DISCUSSION

Benign mechanical obstruction of the common hepatic duct by a stone impacted within the neck or cystic duct of the gallbladder represents an unusual complication of biliary calculi[4]. Indeed, the prevalence of the Mirizzi syndrome at our institution over the last decade was only 0.05% or one in 2000 biliary tract operations for calculus disease. The Mirizzi syndrome has been classified into two types, based on operative and cholangiographic criteria[5,6]. Type I, the acute form, represents extrinsic compression of the common hepatic duct either by the impacted stone within the neck of the gallbladder or cystic duct or by the associated inflammation, as seen in two of our patients. Type II, the chronic form, represents the effects of prolonged compression of the lateral wall of the common hepatic duct by an impacted gallstone, resulting in an erosive cholecystocholedochal fistula or inflammatory structure, as seen in three of our patients. Interbiliary fistulas of the Type II form are rare. Corlette and Bismuth[7] found only 24 such fistulas during a 15-year period involving 3300 biliary operations, and Mallet-Guy[8] noted a 1.1% incidence of gallbladder-to-bile duct fistulas in 3650 biliary operations. Of all internal biliary fistulas, interbiliary fistulas comprise approximately 16%[9].

The anatomic basic of the Mirizzi syndrome has generally been attributed to an anomalous relationship between the cystic duct and common hepatic duct. Dietrich[10] postulated that a low insertion of the cystic duct into the common hepatic duct or, more rarely, a shared mucosal septum between the cystic duct and common hepatic duct contributed to the pathogenesis of the syndrome by predisposing to entrapment of gallstones within this junction. Starling *et al.*[11] similarly emphasized that such anatomic variations provided the focus for intense periductal inflammation, which led to partial mechanical obstruction with jaundice or cholangitis or both. Indeed, these anatomic anomalies are relatively uncommon. Hayes *et al.*[12] described the bile duct anomalies in a series of 400 consecutive primary biliary tract operations, and found anomalies in 189 patients. A low insertion of the cystic duct into the common hepatic duct occurred in 49% of patients, an anomalous junction of the cystic duct with the common hepatic duct in 6%, and a shared mucosal septum in less than 1%. Similarly, Dietrich[10], in a review of 120 consecutive operative cholangiograms, found a low insertion of the cystic duct into the common hepatic duct in 18% of patients, and a common mucosal septum in 2.5%. We found an anomalous relationship between the cystic and common hepatic ducts in two patients herein.

The pre-operative diagnosis of Mirizzi syndrome remains ellusive and requires a high index of suspicion. Clinical history, physical examination and laboratory data are not pathognomonic and only suggest obstructive or cholestatic jaundice. Other conditions such as carcinoma of the gallbladder, carcinoma of the common bile duct or hepatic ducts, and benign stricture of the common bile duct, unassociated with inflammation, may present in similar fashion and must be included in the differential diagnosis. Careful scrutiny of biliary tract imaging should allow for more frequent pre-operative diagnosis of the Mirizzi syndrome and allow differentiation from other pathologic processes.

The salient features of biliary tract imaging that facilitate diagnosis of Mirizzi syndrome are (1) an abnormally thickened gallbladder, (2) large gallstone(s), (3) small eccentric ductal stricture adjacent to the stone, and (4) diffuse proximal bile duct dilatation. Combined use of abdominal ultrasonography and cholangiography probably best define these features. Dewbury[13] described some ultrasonographic features of Mirizzi syndrome that include dilated intrahepatic ducts, and a dilated upper common hepatic bile duct with narrowing just below the porta hepatis at the level of an echodensity characteristic of a calculus. Additionally, ultrasonography may confirm an impacted calculus in the cystic duct or neck of the gallbladder lying at the liver hilus adjacent to the common hepatic duct or its confluence. Ultrasonography in our patients confirmed these findings.

Clemett and Loman[14] described the intravenous cholangiographic findings of Mirizzi syndrome in two patients. They noted a broad curved eccentric compression of the lateral aspect of the common hepatic duct by the stone, with partial duct obstruction, proximal ductal dilatation, and delayed ductal clearing of contrast material. They cautioned that the location of the gallstones may mimic the appearance of choledocholithiasis. Cornud[15] similarly demonstrated the curvilinear compression of the lateral common hepatic duct with partial obstruction and proximal ductal dilatation by transhepatic cholangiography. Moreover, he defined cholangiographically the types of bilio-biliary fistulas associatede with Type II Mirizzi syndrome: cholecysto-hepatic duct fistulas and cystic duct-hepatic duct fistulas. A cholecysto-hepatic duct fistula can occur between the gallbladder and common hepatic duct, right hepatic duct, or even the left hepatic duct at the confluence. In this situation, identification of the cystic duct may not be discernible because of the inflammatory reaction. A cystic duct-hepatic duct fistula tends to occur between the cystic duct or neck of the gallbladder and common hepatic duct. Interestingly, interbiliary duct fistulas are almost always produced by benign rather than malignant conditions[7].

The surgical management of the Mirizzi syndrome requires precise definition of the biliary tract pathology. Thus, accurate pre-operative imaging of the biliary tract will not only facilitate intra-operative dissection of the chronically inflamed and often fragile ductal system, but will often indicate the presence of a fistula, its location, or the need for bilioenteric bypass.

Ideally, cholecystectomy and common duct exploration are the integral components of surgical therapy. Cholecystectomy removes the primary pathology—gallstones—and provides exposure to assess associated abnormalities of the common hepatic or bile duct. Acute and chronic pericholecystic inflammation technically may complicate cholecystectomy and increase operative risk. Because of the intense inflammation in the triangle of Calot and the frequency of anatomic bile duct anomalies and interbiliary fistula, dissection for cholecystectomy should proceed from the fundus toward the cystic duct. In fact, we have found that cholecystotomy or partial cholecystectomy and extraction of gallstone facilitates assessment of the site of obstruction of the common duct and operative cholangiography. Moreover, this approach preserves the cystic duct or Hartman's pouch, allowing for autogenous biliary mucosal repair of the interbiliary fistula. If pre-operative cholangiography has not been performed, operative cholangiography is imperative to detect potential biliary fistula or bile duct stones and to confirm the integrity of the remaining biliary ductal system[7].

Common bile duct exploration is recommended to remove common duct stones and to assess the degree and severity of the inflammatory stricture, unless the inflammatory reaction is so great that a safe exploration is precluded. Cholangiography should precede exploration, to obviate negative explorations that cause significant risk in patients with the Mirizzi syndrome. Prior to choledochotomy, the entire extrahepatic duct, from the ductal confluence to the duodenum, must be visualized to avoid injury to ductal anomalies. The proximal bile duct is best utilized for choledochotomy if possible. Choledochotomy in the dilated bile duct, rather than distally, will allow for easier stone extraction, instrumentation for assessing the severity of the stricture, and biloenteric bypass if necessary. If intraoperative pathology mimics cholangiocarcinoma, biopsy can be performed either directly or through the choledochoscope. In patients with interbiliary fistula, the common hepatic duct often can be repaired with a rim of gallbladder, or the remnant of gallbladder can be anastomosed to the duodenum, creating a cholecystocholedochoduodenostomy[16]. Alternative methods of repair of non-circumferential bile duct defects utilizing the duodenum or a Roux-Y limb of jejunum have also proven successful[17]. Rarely, a Roux-Y hepaticojejunostomy may be required with luminal compromose after direct interbiliary fistula closure or rigid inflammatory strictures. T-tube choledochostomy provides access to the ductal system until complete resolution of the inflammation.

## References

1. Kehr, H. (1905) *Die in neiner klinik geubte technik der gallensteinoperationen mit einen hinweis auf die indikationen und die dauerersolge*. Munich J.F. Lehmann
2. Ruge, E. (1908) Dietrage zur chirurgischen anatomie der grossen gallenwege (ductus hepaticus, choledochus, und pancreaticus). *Arch. Clin. Chir.*, **LXX V–II**, 47
3. Mirizzi, P.L. (1948) Sindrome del conducto hepatico. *J. Int. Chir.*, **8**, 731
4. Lubbers, Evert-Jan C. (1983) Mirizzi syndrome. *World J. Surg.*, **7**, 780–785
5. McSherry, C.K., Ferstenberg, H. and Virshup, M. (1982) The Mirizzi syndrome: suggested classification and surgical therapy. *Surg. Gastroenterol.*, **1**, 219–225
6. Morelli, A., Narduci, F. and Ciccone, R. (1978) Can Mirizzi syndrome be classified into acute and chronic form? *Endoscopy*, **10**, 109–112
7. Corlette, M.B. and Bismuth, H. (1975) Bibliobiliary fistula. A trap in the surgery of cholelithiasis. *Arch. Surg.*, **110**, 377–383
8. Mallet-Guy, P. and Roget, C. (1960) Les fistules cholecystocholedocrines. *Lyon. Chir.*, **56**, 231–245
9. Kourias, P. and Tsoufis, E. (1959) Fistuls biliaries internes spontanees d'origine lithiasique. *J. Chir. (Paris)*, **75**, 353–374
10. Dietrich, K.F. (1963) Die hepatikussenose bei gallenblasenhals und zystikusstienen (Mirizzi syndrome). *Bruno. Beitr. Klin. Chir.*, **206**, 9
11. Starling, J.R. and Matallana, R.H. (1980) Benign mechanical obstruction of the common hepatic duct (Mirizzi syndrome). *Surgery*, **88**(5), 737–740
12. Hayes, M.A. Goldenberg, I.S. and Bishop, C.C. (1958) The developmental basis for bile duct anomalies. *Surg. Gynecol. Obetstrics*, **107**, 447–455
13. Dewbury, K.C. (1979) The features of the Mirizzi syndrom on ultrasound examination. *Br. J. Radiol*, **52**, 990–992
14. Clemett, A.R. and Loman, R.M. (1965) The roentgen features of the Mirizzi syndrome. *Am. J. Roentgenol.*, **94**, 480
15. Cornud, F., Girenier, P., Belghiti, J. *et al.* (1981) Mirizzi syndrome and biliobiliary fistulas: Roentgenologic appearance. *Gastrointest. Radiol.*, **6**, 265–268
16. Blumgart, L.H. (1988) Editor. *Surgery of the Liver and Biliary Tract*, pp. 721–752. London: Churchill Livingstone.
17. Rutledge, R.H. (1983) Methods of repair of noncircumferential bile duct effects. *Surgery*, **93**, 333–342

*Accepted by L. Blumgart on 8 January 1988*

## INVITED COMMENTARY

Modern imaging techniques have put us in a position to obtain in a few days time a precise anatomical delineation of the cause of obstructive jaundice. Usually the anatomical image allows for a putative pathological diagnosis as well. By far the most frequent cause of primary obstruction (i.e. without antecedent surgery) is cancer. The dismal prognosis of hepatic duct cancer often leads to abstention from surgery and palliation by intubation.

The merit of the communication of Bower and Nagorney is that it accentuates again the small but real possibility that lesions of the hepatic ducts and their confluence may be caused by gallstone disease and thus be amenable to curative surgery.

Admittedly, gallstones as a cause of hepatic duct stenosis remain a rarity. However, in an ageing population acquired diseases such as gallstones increase in relative importance. Three out of five patients reported in this paper were over 65 years of age. Therefore, we all need to be familiar with the possible complications of cholecystolithiasis. Also, these observations strengthen the case for histological verification of hepatic duct lesions whenever this is possible without undue hazard.

The diagnosis of a possible benign lesion was made preoperatively only once in these patients, illustrating the difficulty of predicting histology on the basis of X-ray pictures. In this respect, the authors have collected some useful details which may be helpful.

Even more important than a preoperative pathological diagnosis is, as it seeems to me, a precise anatomical representation of the lesion. In this respect I would have liked to have seen discussed the possible contribution of ERCP. There is a useful discussion of surgical options once the anatomical situation is demonstrated. I do not understand the fear for negative explorations of the common duct. I would think that the treatment of the lesion under discussion aims at two things: eradication of the cause (i.e. gallstones) by cholecystectomy, and relief of obstruction of the duct. Only in cases of minimal inflammation (case 4?) could exploration possibly be called negative. In all other cases exploration and T-tube drainage is necessary, as the authors rightly stress in the last sentence of their paper.

All in all, it is a useful paper which I have enjoyed reading. There remains the question of the title. Mirizzi described the condition that has been given his name as a functional disturbance of a sphincter in the hepatic duct, triggered by several possible causes amongst which, gallstones. Conclusive evidence for the existence of this sphincter has never been found and the concept of a hepatic sphincter has disappeared from textbooks and journals. With the disappearance of its bore — a functional disturbance of a sphincter — the Mirizzi syndrome has ceased to exist.

Further, the impact of gallstones upon the hepatic ducts varies enormously in extent and in severity, producing a wide spectrum of damage from simple displacement to bioliobiliary fistulas and stenosing cholangitis. The present paper contains examples of this variation: strictly speaking case 4 does not even come under the definition used. What all these conditions have in common is a) the cause (gallstones) and b) the histology (benign).

I admit that no useful purpose is being served by retaining the name of a non-existing syndrome to designate a wide range of pathological conditions, the consequences of which may vary fom innocuous to severe liver damage. What should be retained is the notion that among a large majority of malignant stenoses of the bile duct we should go on looking for the occasional benign one in order to protect our patients for the mishap of a wrong diagnosis!

E.J.C. Lubbers,
Nijmegen, The Netherlands
